# Microfluidic paper device for rapid detection of aflatoxin B1 using an aptamer based colorimetric assay†

**DOI:** 10.1039/d0ra00062k

**Published:** 2020-03-24

**Authors:** Aruna Kasoju, Narlawar Sagar Shrikrishna, Deepshikha Shahdeo, Azmat Ali Khan, Amer M. Alanazi, Sonu Gandhi

**Affiliations:** DBT-National Institute of Animal Biotechnology Hyderabad-500032 India sonugandhi@gmail.com gandhi@niab.org.in; Department of Biotechnology, JNTUA College of Engineering Andhra Pradesh-516390 India; Department of Pharmaceutical Chemistry, College of Pharmacy, King Saud University Riyadh-11451 Saudi Arabia

## Abstract

Contamination of milk by mycotoxins is a serious problem worldwide. Herein, we focused on the detection of aflatoxin B1 (AflB1) using a paper microfluidic device fabricated with specific aptamers as biorecognition elements. The fabrication process resulted in the generation of a leak proof microfluidic device where two zones were designed: control and test. Detection is achieved by color change when aflatoxin reacts with an aptamer followed by salt induced aggregation of gold nanoparticles. Specific aptamers for aflatoxin B1 were immobilized successfully onto the surface of gold nanoparticles. Biophysical characterization of the conjugated AuNPs–aptamer was done by UV-vis spectroscopy, DLS (dynamic light scattering), TEM (transmission electron microscopy). Under optimal conditions, the microfluidic device showed an excellent response for aflatoxin B1 detection in the range of 1 pM to 1 μM with a detection limit of up to 10 nM in spiked samples. Disadvantages associated with conventional techniques of ELISA were overcome by this one step detection technique with low operation cost, simple instrumentation, and user-friendly format with no interference due to chromatographic separation. The developed microfluidic paper-based analytical device (μPAD) can provide a tool for on-site detection of food toxins in less than a minute which is the main requirement for both qualitative and quantitative analysis in food safety and environmental monitoring.

## Abstract 2

Contamination of milk by mycotoxins is a serious problem worldwide.

## Graphical abstract

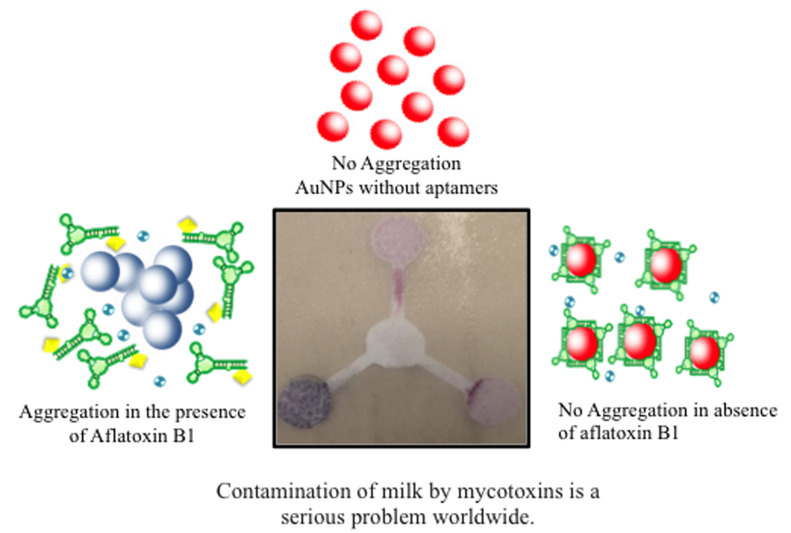

## Introduction

Aflatoxin contamination of milk is very common and has been classified as a group 1 carcinogen.^1^ Mycotoxins are produced as secondary metabolites by fungus *Aspergillus* and *Penicillium* species. It enters into an animal's body through animal feed contaminated with aflatoxin or crops contaminated before harvest. Animal products such as milk serve as the major source of aflatoxins which indirectly become responsible for increased mortality of humans as well as farm animals. The maintenance of milk quality is of immense importance due to its large scale consumption worldwide. Contamination of milk with aflatoxin B1 poses serious health issues in humans as well as in animals such as damage to the reproductive system, necrosis of tissues, potent carcinogenesis, organ lesions, mutagenesis, nervous system failure, decrease in immune function, and even death.^2,3^

Currently available methods for detection of aflatoxins are cumbersome, less sensitive, require expensive instrumentation, and cannot rapidly detect aflatoxin B1 immediately on field. The present investigation was undertaken with a view to overcome these shortcomings. Aflatoxins possess significant ultraviolet (UV) absorption and fluorescence properties that in combination with chromatographic methods, either high performance liquid chromatography (HPLC) or thin layer chromatography (TLC), are widely used in detection.^4^ Such methods require sample extraction and extract clean-up by solid-phase extraction (SPE) or immunoaffinity chromatography (IAC) followed by chromatographic separation.^5–7^ Liquid chromatography-mass spectrometry (LC-MS) technology offers the advantage of “dilute and shoot” techniques where sample extracts are analyzed without clean-up, and with the added advantage of multi-mycotoxin analysis whereby a range of mycotoxins can be analyzed in the same sample analysis but LC-MS requires trained personnel and laboratory set up.^8,9^ Available methods of analysis range from, on-field rapid diagnostic strips such as airstrips used in rapid test kits based on competitive enzyme-linked immunosorbent assay (ELISA) with colorimetric detection to spectroscopic methods.^10,11^ Recently graphene oxide and gold nanowires were used as novel nanomaterials to develop FRET and electrochemical based sensors for detection of aflatoxin.^12–14^ Complete elimination of mycotoxin contamination in agricultural products is not possible because contamination can occur anywhere in the food chain from harvest to feeding and environmental factors also play a major role.^15^ Hence, it is very important to develop detection tools or biosensors with high sensitivity and selectivity for on-site monitoring of aflatoxins in animal feed and milk. Biosensors are devices used to detect the presence or concentration of a biological analyte, such as narcotic drugs, pesticides, or infectious disease biomarkers.^3,16–24^ To meet this requirement, paper based microfluidic devices have gained importance. Recently, microfluidic paper-based analytical devices (μPAD) have been demonstrated as a low-cost alternative to glass and polymer substrates for fabrication.^25,26^

Patterning paper with hydrophobic barriers allows confinement of liquid to well-defined hydrophilic regions, and fluid flow *via* capillary action.^27^ Paper is an attractive choice for developing microfluidic devices because of widely available matrices made-up of extremely cheap cellulosic material, good compatibility with chemicals/biochemicals used in bio-medical applications, and easy movement purely due to capillary forces ensuring no external devices are needed to enable the flow of the reagent or analyte in the channels.^28,29^ A variety of 2D and 3D microfluidic channels on paper substrates that are capable of transporting liquids in predesigned pathways have been reported in literature.^30^

In this current study, we have reported the detection of aflatoxin B1 using specific aptameric–gold nanoparticles (Afl B1–Apt–AuNPs) nanoconjugates on a microfluidic paper-based analytical device (μPAD). The Apt–AuNPs nanoconjugate was adsorbed onto the paper *via* physical adsorption and Afl B1 was allowed to flow over the μPAD. The biophysical characterization of nanoconjugate was done by UV-vis spectroscopy, DLS (dynamic light scattering) for hydrodynamic diameter and zeta potential measurements, and TEM (transmission electron microscopy). The sensitivity of the assay was measured by spectroscopic and capillary methods using μPAD. For this, aflatoxin B1 was detected in the range of 1 μM to 1 pM, with a detection limit of up to 10 nM in standard samples, making it suitable to detect on field with spectroscopic method upto 10 nM. The fabricated μPAD is sensitive, cost effective, and can be made applicable for on-site monitoring after integration into an electronic device for real time detection.

## Materials and methods

Gold(iii) chloride aflatoxin B1 (Afl B1), and ochratoxin were purchased from Sigma-Aldrich. Sodium citrate and sodium chloride were procured from Sisco Research Laboratories Pvt. Ltd. (SRL), India. The aptamer sequence of Afl B1 was GTTGGGCACGTGTTGTCTCTCTGTGTCTCGTGCCCTTCGCTAGGCCCACA (5′ to 3′). The ssDNA oligonucleotides were synthesized by GCC biotech. Stock solutions (100 μM) of aptamers were dissolved in ultrapure water and stored at −20 °C. Whatman filter paper was purchased from GE healthcare, India. Trysulfonium hexaflurophosphate and propylene glycol monomethyl ether acetate (PGMEA) were procured from Sigma-Aldrich. All reagents were of analytical grade and used as received.

### Synthesis and characterization of gold nanoparticles

Gold nanoparticles (AuNPs) were synthesised as per the standard Turkevich protocol.^31^ Gold chloride (0.01 mL, 10%) was added to Milli-Q water in an Erlenmeyer flask. Mixture was stirred using a magnetic stirrer and heated to boiling temperature using hot plate. 1 mL of 1% sodium citrate was rapidly added to the boiling gold chloride solution, which resulted in gradual change in the colour of solution from yellow to blue and finally to wine red. The reaction was stopped after 20 min and particles were cooled and stored at 4 °C until further use. The synthesized AuNPs size was 19 nm with UV-vis absorption peak at 520 nm. The Afl B1 aptamers were incubated with AuNPs to confirm the adsorption on its surface. For this, 1 mL of AuNPs solution was incubated overnight with 800 nM of Afl B1 at 4 °C and centrifuged at 10 000 rpm at 4 °C. The AuNPs–Afl B1 was finally dissolved in 50 μL and used directly for TEM. Gold nanoparticles (AuNPs and AuNPs–AflB1 apt) were characterized using UV-vis spectroscopy, DLS, and TEM.

### Aggregation assay for optimization of NaCl concentration

The aggregation assay was optimized, where sodium chloride (NaCl) was prepared at variable concentrations (20, 40, 80, 160, 320, 640 mM) and incubated with a fixed concentration of AuNPs. The AuNPs–NaCl complex was further characterised by UV-vis spectroscopy. Ratio of absorbance at 520 nm and 630 nm was taken to know the optimum concentration of NaCl inducing aggregation, and DLS (hydrodynamic size, zeta potential).

### Optimization of Afl B1–aptamer concentration

Aflatoxin B1 aptamer was adsorbed on the surface of AuNPs *via* physical adsorption. Aptamers (Afl B1 and ochratoxin) were prepared at different dilutions (25, 100, 200, 400, 800 nM) in 1× PBS, pH 7.4 and incubated for 10 min with a fix concentration of AuNPs. Fixed concentration of NaCl (160 mM) was added to each dilution and further incubated for 2 min. The resulting solutions were observed for the change in the colour gradient from red wine to blue to grey. Similar characterization techniques were used as stated in the above section and a calibration curve was plotted using the ratio of absorbance at 520 nm and 630 nm to observe the process of aggregation. The fluorescence intensity was measured with excitation at 360 nm and emission spectra was collected in the range of 418 to 440 nm.

### Development of paper device for aflatoxin B1 detection

To fabricate the device 52% w/w photoresist was dissolved in 5% v/v trysulfonium hexaflurophosphate and 43% v/v propylene glycol monomethyl ether acetate. Resin mix was then allowed to mix properly and spread uniformly on the surface of Whatman filter paper and allowed to dry for 5 min at room temperature (RT). Further the dried paper was baked till complete evaporation of the solvent. Photomask was kept on the top of the dried resin coated paper and further exposed to UV light for 2 min followed by baking for 10 min. Paper was washed with acetone and allowed to dry at RT. Afl B1 aptamers were allowed to get adsorbed onto the surface of AuNPs as stated previously. Aflatoxin B1 serial dilutions were prepared in the range of 1 μM to 1 pM in Milli-Q water and milk and incubated for 2 min with a fixed concentration of NaCl (160 mM) and characterized using UV-vis spectroscopy, and DLS (hydrodynamic diameter and zeta potential). The AflB1–Apt/AuNPs–NaCl nanoconjugates were allowed to react with free aflatoxin B1 *via* displacement assay in solution phase (vials) as well on μPAD.

## Results and discussions

### Aggregation assay

**Scheme 1:**
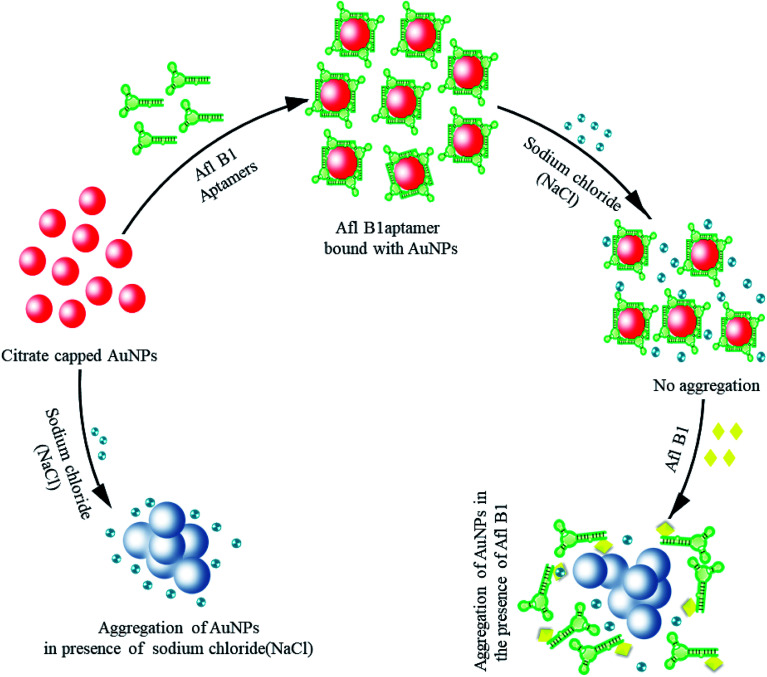
Colorimetric assay for the detection of aflatoxin B1 (Afl-B1) using salt induced AuNPs aggregation. In the absence of aflatoxin B1, aptamer adsorbed on the surface of *via* physical adsorption and enhanced the stability against salt (NaCl) induced aggregation by formation of G-quadruplex. In presence of aflatoxin B1, aptamer bound with AuNPs displaced and form a complex with aflatoxin B1 leaving AuNPs to react with NaCl that resulted in aggregation (change in color from wine red to blue).

Gold nanoparticles (19 nm) were prepared by citrate reduction method.^32–35^ Aggregation of gold nanoparticles is due to strong van der Waals forces at high salt concentrations that alter the charges on the surface of AuNPs resulting in aggregation.^36^Scheme 1 depicted colorimetric method for Afl B1 aptasensing. In this method, monodispersed gold colloids were prepared *via* citrate reduction method with SPR peak at 520 nm. The Afl B1 aptamers adsorbed on the surface of AuNPs *via* physical adsorption. In the presence of electrolytes (NaCl), the charge repulsion between particles gets reduced, resulting in aggregation. This aggregation caused an increase in absorbance at *A*_630_ along with change in the colour of AuNPs from red wine to blue. In the presence of a specific analyte Afl B1, the aptamer dissociated from AuNPs after binding with the analyte molecule (AFL B1) and changing their conformation to G-quadruplex, which once again resulted in the aggregation of unprotected AuNPs upon the addition of NaCl.

### Characterization of NaCl induced AuNPs complex

**Fig. 1:**
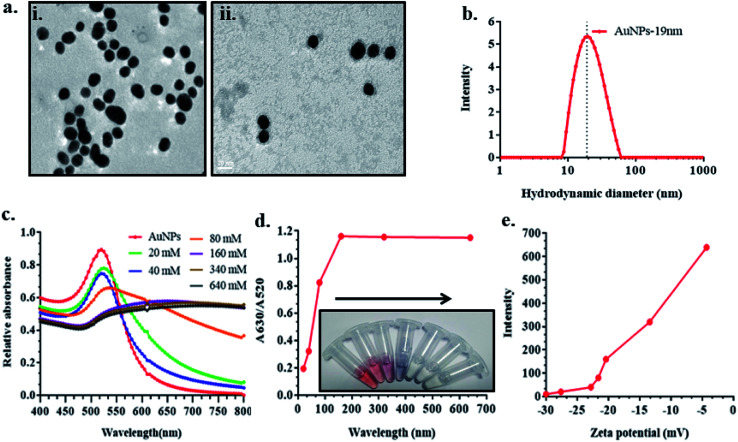
Optimization of NaCl concentration for the detection of aflatoxin B1. (a) TEM showed the (i) average AuNPs size of 19 ± 5 nM (ii) AuNPs coated with AflB1 aptamer; (b) DLS spectra showed hydrodynamic diameters of 19 nm; (c) UV-vis spectroscopy at different concentration of NaCl (20, 40, 80, 160, 340, 640 mM) showed the red shift from 520 nm (AuNPs) to 525 nm (AuNPs–NaCl complex), as the concentration of NaCl increased, the spectra became flattened; (d) calibration curve and corresponding color change w.r.t. optimum concentration of NaCl; (e) zeta potential of similar concentration of NaCl reacted with AuNPs.

The gold nanoparticles of 19 ± 5 nM were synthesized and confirmed with TEM and DLS (Fig. 1a(i) and b). The morphological features indicated monodispersed particles with uniform size distribution. Fig. 1a(ii) confirmed the deposition of Afl B1 aptamer on the surface of AuNPs without causing any aggregation as the gold nanoparticles observed in monodispersed state. The presence of NaCl led to aggregation of AuNPs. Fig. 1c shows the absorption spectra of AuNPs in the presence and absence of NaCl. Different concentrations of NaCl (20, 40, 80, 160, 340, 640 mM) were added to know about the optimum concentration that led to aggregation of AuNPs. It was observed that AuNPs were stable at low concentrations of salt that makes the application less suitable for detection of aflatoxin. The calibration curve was plotted and optimum concentration observed for aggregation was 160 mM. As shown in Fig. 1d, the absorbance intensity reached its maximum at 160 mM where the color developed was most obvious. The zeta potential was found to have increased (−30, −28, −23, −21, −20, −12, −5 mV) due to increase in salt concentration (Fig. 1e). The confirmation of aptamer in saline buffer changed antiparallel to a G-quadruplex structure, that allowed the aptamer to bind with the targeted toxin (Afl B1) and gold nanoparticles were no longer protected at this stage, which resulted in salt induced aggregation and thus the color changed from red to blue.

### Effect of aflatoxin B1 aptamer concentration on AuNPs

**Fig. 2:**
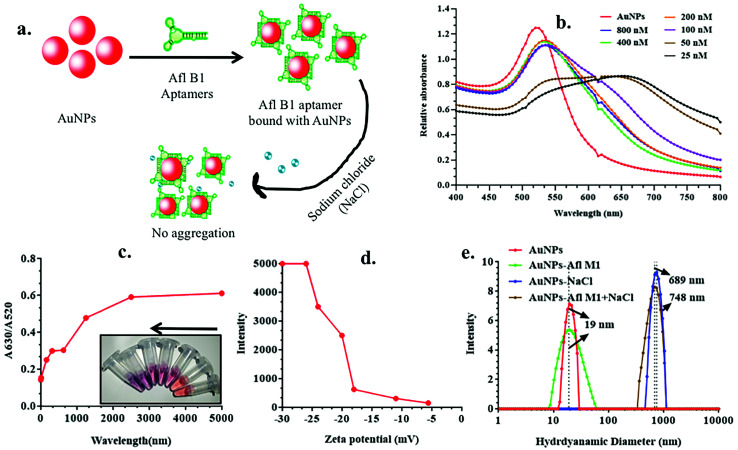
a) Diagrammatic representation for optimization of aptamer concentration for the detection of aflatoxin B1; (b) absorption spectra of different concentration of aptamer (800, 400, 200, 100, 50, 25 nM); (c) calibration curve for detection of optimum concentration of aptamer (25, 50, 100, 200, 400, 800 nM) and correspondence color change in the presence of different concentration of aptamer; (d) zeta potential of different concentration of aflatoxin B1 aptamer; (e) hydrodynamic diameter of AuNPs, AuNPs + AflB1 Apt, AuNPs + NaCl, and AuNPs–AflB1 + NaCl nanocomplex.

Schematic representation of experimental condition in Fig. 2a was correlated with the absorption spectra obtained in Fig. 2b, where fixed concentrations of AuNPs and NaCl were added to variable concentrations of Afl B1 aptamer (25, 50, 100, 200, 400, 800 nM). A red shift as well decrease in absorbance spectra were observed if when compared with only AuNPs. At 25 and 50 nM concentration of Afl B1, the peak was almost flattened that indicated less number of aptamers in solution to prevent the process of aggregation. Fig. 2c showed the ratio of the absorbance at 630 and 520 nm that confirmed the AuNPs–NaCl/aptamer stability after 200 nM. Fig. 2d depicted the increase in the zeta potential (−30, −25, −22, −20, −17, −11, −5 mV) with increasing concentration of Afl B1 aptamers. The hydrodynamic diameter was also analyzed to confirm the aptameric adsorption on the surface of AuNPs in the presence of NaCl (Fig. 2e). The hydrodynamic diameter of AuNPs and AuNPs–Afl B1 was found to be 19 nm, which after addition of NaCl increased to 689 nm, and with further addition of Afl B1, the size increased to 748 nm, which reconfirmed the binding process. The concentration of aptamer is the key to delineate the sensitivity of the assay. Less concentration of aptamers cannot stop the process of AuNPs aggregation with any given amount of NaCl due to insufficient number of aptamers adsorbed onto the surface of AuNPs which does not completely protect the AuNPs. Increase in the aptamer concentration might have led to decrease in the sensitivity because most of the aptamer will remain in the unbound state. Therefore, optimum concentration of aptamer is the pre-requirement to stop the aggregation of AuNPs as well as maintain sensitivity of the assay.

### Analytical performance for the detection of aflatoxin B1

**Fig. 3:**
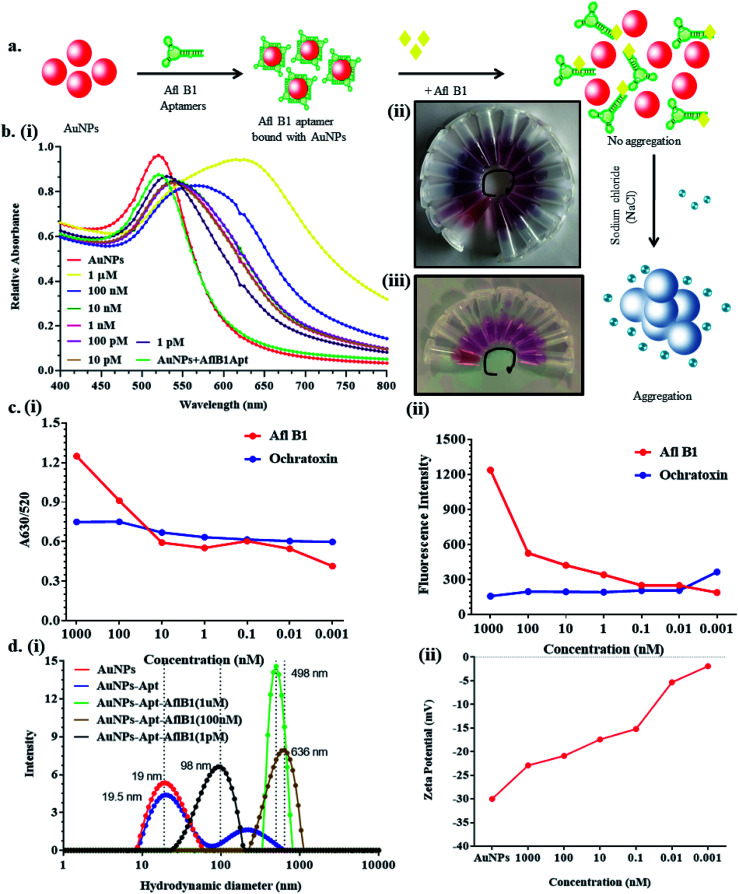
a) Scheme showed the step by step displacement assay; (b) (i) UV-vis spectroscopy of AuNPs–Apt complex in the presence and absence of aflatoxin B1 at different concentrations (1 μM to 1 pM) wit fix concentration of NaCl and (ii and iii) corresponding change in color at various concentration of aflatoxin B1 and ochratoxin (1 μM-blue to 1pM-wine red); (c) (i) calibration curve at A630/520 and (ii) fluorescence spectra of Afl B1 and ochratoxin; (d) (i) Hydrodynamic diameter and (ii) zeta potential of AuNPs, AuNPs + Apt, AuNPs + Apt + AflB1 nanocomplex.

**Fig. 4:**
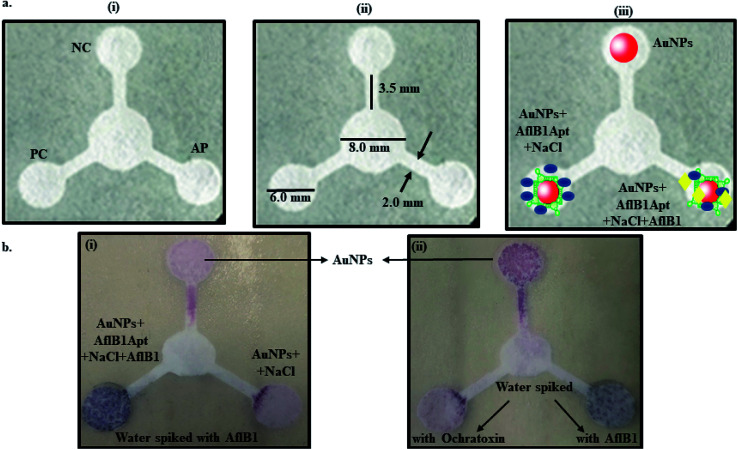
Schematic representation of μPAD paper microfluidic device. (a) (i) Concept of μPAD-positive control (PC), negative control (NC), analyte pad (AP), (ii) dimensions and (iii) fabrication of the paper device; (b) (i) the color development after spiking Afl B1 in water and (ii) color development in presence of AflB1/no change in color in presence of ochratoxin in water.

Fig. 3a depicted schematic representation of competitive inhibition assay in the presence of Afl B1. Absorption spectra of competitive assay was in complete agreement with the figure shown. The red shift was obvious at 1 μM Afl B1 (blue color), while at 100 nM and 10 nM Afl B1 concentrations, the color changed to reddish blue (Fig. 3b). The absorbance decreased with red shift and similar pattern in fluorescence intensity was observed when decreasing concentration of Afl B1 was added, indicated less displacement of aptamers due to low concentration of free molecules (Fig. 3c(i and ii)). There was no change in absorbance and fluorescence intensity when ochratoxin was replaced with aflatoxin B1 (Fig. 3c(i and ii)). Fig. 3d(i) showed the change in hydrodynamic diameter of AuNPs from 19 nm to 19.5 nm (AuNPs–Apt), 498 nm (AuNPs–Apt/Afl B1 (1 μM) + NaCl), 636 nm (AuNPs–Apt/Afl B1 (100 nM) + NaCl), and 98 nm (AuNPs–Apt/Afl B1 (1 pM) + NaCl). Change in zeta potential was also observed as shown in Fig. 3d(ii). As the concentration of Afl B1 decreased, zeta potential increased from −30 to −1.9 mV. The fabricated paper microfluidic device as shown in Fig. 4a(i and ii), had dimensions 6 mm of μPAD, 3.5 mm length, 2 mm thickness of the microchannel. The samples spiked on the sample pad developed color immediately after application (Fig. 4b(i)). It was observed that in case of AuNPs (negative control) and AuNPs–NaCl complex, there was no change in color, while in case of and AuNPs–Apt/Afl B1 + NaCl (positive control), the color was changed immediately to blue (Fig. 4b(ii)). The specificity for Afl B1 aptamer was also analyzed. For this purpose, ochratoxin was spiked in water and applied directly on μPAD and no change in color was observed. We prepared aflatoxin B1 in the range of 1 μM to 1 pM and the detection limit in this case was found to be 10 nM, if compared with ELISA IC_50_ was found to be 1 nM (Fig. S1†). The developed device is quite handy and portable, and can be carried to any place for immediate analysis for the presence of aflatoxins in animal feed or milk samples.

## Conclusion

In summary, we have developed two different methods for the detection of Afl B1. First, a spectroscopic and second a μPAD device based method. The spectroscopic method can detect upto 100 nM, whereas the μPAD device showed a detection limit upto 10 nM. μPAD is a label free, simple, rapid, and sensitive assay developed for the detection of aflatoxin B1 using aptamer–AuNPs nanoconjugates. This paper based colorimetric method determined the presence of aflatoxin B1 with a simple displacement assay of AuNPs without the requirement of sophisticated instrumentation (in case of spectrophotometry) and expensive methods. The change in the color from red to blue can be easily distinguished with naked eyes or by UV-vis spectroscopy. The standardization of NaCl, aptamer, and aflatoxin concentrations was done to determine the detection limit which was approximately 10 nM in standard samples. The food industry could be greatly benefited from this proposed inexpensive device, as this analytical technique could be used to detect aflatoxin B1 using aptamer as the biorecognition element, and AuNPs as an indicator on paper microfluidic device. Further, we are exploring the use of patterned-paper as a platform for multianalyte detection with an aim of developing low-cost diagnostic devices.

## Conflicts of interest

There is no conflict of interest among all authors of the papers.

## Supplementary Material

RA-010-D0RA00062K-s001
