# Supplementary material for: Microfluidic paper device for rapid detection of aflatoxin B1 using an aptamer based colorimetric assay
Source: RSC Adv. 2020 Mar 24;10(20):11843–50. doi: 10.1039/d0ra00062k (PMC9050516; doi:10.1039/d0ra00062k)
Supplement: RA-010-D0RA00062K-s001 [file RA-010-D0RA00062K-s001.pdf]

Electronic Supplementary Material (ESI) for RSC Advances.
This journal is © The Royal Society of Chemistry 2020

Microfluidic Paper Device for Rapid Detection of Aflatoxin B1 using Aptamer Based Colorimetric Assay

Aruna Kasoju[a,b], Narlawar Sagar Shrikrishna[a*], Deepshikha Shahdeo[a*], Azamat Ali Khan[c], Amer M. Alanazi[c], Sonu Gandhi[a,#]

[a]DBT-National Institute of Animal Biotechnology, Hyderabad-500032, India

[b]Department of Biotechnology, JNTUA College of Engineering, Andhra Pradesh- 516390, India

[c]Department of Pharmaceutical Chemistry, King Saud University, Riyadh-11451, Saudi Arabia

[#]Correspondence:

Dr. Sonu Gandhi; E-mail: sonugandhi@gmail.com; gandhi@niab.org.in

[*]Deepshikha Shahdeo and Narlawar Sagar Shrikrishna contributed equally to this work.

Running head: Microfluidic device for aflatoxin B1 detection

Materials and Methods:

Enzyme Linked Immunosorbent Assay.

96 well Nunc ELISA plate was coated with 0.5 µg/mL Afl B1 mouse monoclonal antibody (100 µL/well) in 0.1 M carbonate buffer, pH 9.6 and incubated overnight at 4 °C. Blocking was done with 5% BSA in 1x PBS, pH 7.4 for 2 h at 37 °C. Afl B1 (0.01 nM to 1 µM) and HRP-conjugated AflB1 (1:100, fixed) was prepared in 1xPBS, pH 7.4 and 100 µL/well was added for competitive reaction for 1h at 37 °C. TMB substrate (100 µL/well) was added and incubated further for 15 min at RT followed by addition of (50 µL/well) stop solution. Washing was done three times with 1x PBS-T (Tween 20), pH 7.4 after completion of each step. The intensity of the color is directly proportional to the amount of bound conjugate and inversely proportional to the concentration of aflatoxin in the sample or standard. Therefore, as the concentration of aflatoxin in the sample or standard increases, the intensity of the blue color will decrease.

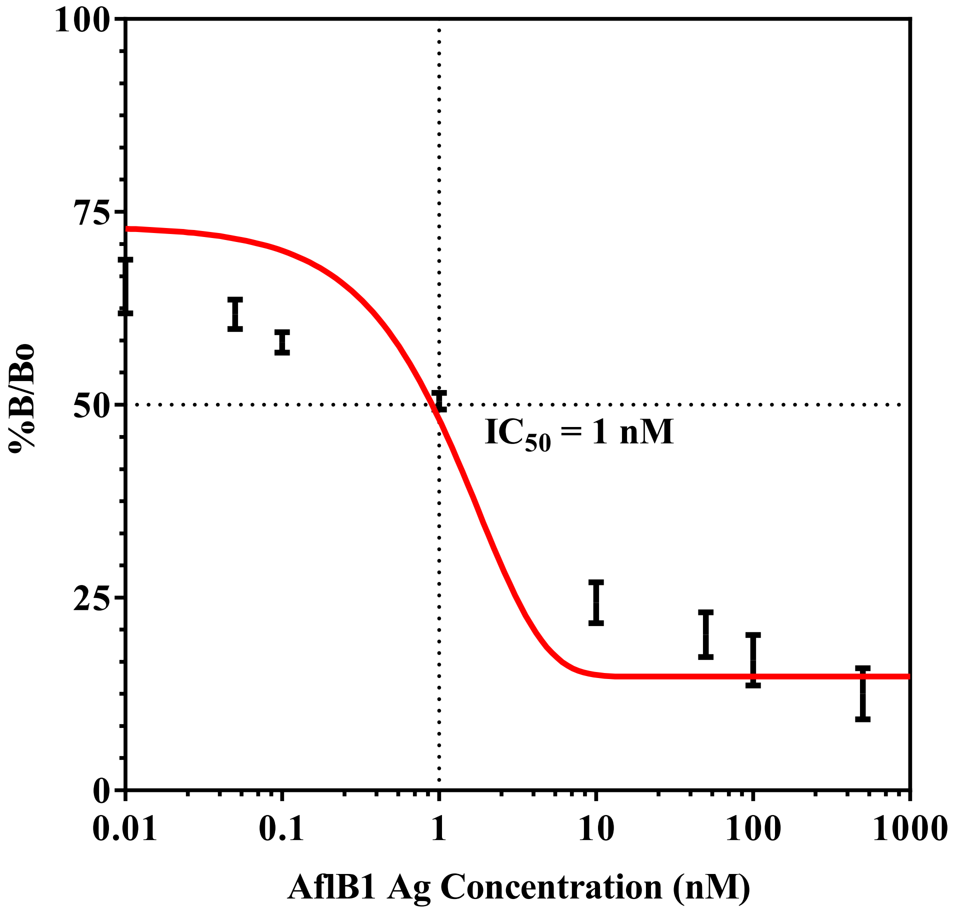

Fig. S1. Competitive inhibition assay using AflB1 antibody (0.1 μg/mL) for coating. The concentration of HRP-AflB1 conjugate was kept at 1:100, while AflB1 antigen was prepared in the range of 0.01 nM to 1 μM.
